# A systematic review of antimicrobial resistance transmission inferences at the human-livestock interface in Africa

**DOI:** 10.1038/s44259-025-00126-y

**Published:** 2025-06-30

**Authors:** Frank Chilanga, Keneth I. Kasozi, Stella Mazeri, Gavin K. Paterson, Adrian Muwonge

**Affiliations:** 1 The Digital One Health Laboratory, Royal (Dick) School of Veterinary Studies and The Roslin Institute, Easter Bush Campus, Midlothian, UK; 2 Infection Medicine, Deanery of Biomedical Sciences, Edinburgh Medical School, College of Medicine and Veterinary Medicine, The University of Edinburgh, Edinburgh, UK; 3 Royal (Dick) School of Veterinary Studies and The Roslin Institute, Easter Bush Campus, Midlothian, UK

**Keywords:** Medical research, Epidemiology, Bacteria, Microbiology, Antibiotics

## Abstract

The transmission of antibiotic-resistant bacteria across multi-species networks is a contributor to the global challenge of antimicrobial resistance (AMR). AMR transmission inferencing, a retrospective process, is critical for refining the evidence underpinning current control strategies. In Africa, where AMR is associated with an estimated 1.05 million deaths annually, it is crucial to evaluate how AMR transmission inferences are made and to consider their implications for achieving national action plan goals. Key questions that need to be addressed in these settings include: (a) How is transmission defined? (b) How are transmission studies designed? (c) Which pathogens or commensal bacteria are used to infer AMR transmission? (d) How granular and reliable is the data used to make transmission inferences? (e) Can the frequency of transmission events be quantified? and (f) Can the directionality of transmission between hosts be established? In this systematic review, we examine the evidence informing current control strategies by analysing 34 studies from Africa, involving 18,604 human and livestock samples and 16 sentinel bacteria. Transmission inferences largely rely on cross-sectional studies with limited sample representativeness. Gram-negative bacteria, mainly *Escherichia coli* (64.71%), form the basis of most inferences. Most inferences remain qualitative, and analyses often overlook uncertainty quantification. In addition, studies are potentially underpowered as only 25% of collected samples are used for transmission inferencing. Based on this analysis, we propose a framework that leverages the growing use of genomic epidemiology to infer AMR transmission with an aim of supporting the design and evaluation of targeted interventions.

## Introduction

Antimicrobial resistance (AMR) occurs when pathogens no longer respond to the antimicrobials administered to kill them or inhibit their growth. This definition is biased toward clinical settings, i.e., medical or veterinary, whereas in the environment, AMR is a natural defence in microbial ecology. The emergence of AMR is well-studied, with pathways primarily driven by selection pressure driven by antibiotic use^1–3^. The effects of AMR on global public health^4,5^ and food safety^6^ directly and indirectly contribute to poor community health outcomes, particularly the loss of lives in developing countries^4^. AMR is driving healthcare costs beyond the reach of millions of people^7^. Without the reduction of AMR transmission, the associated and attributable deaths, particularly among infants and the elderly, are expected to increase^8^.

**Fig. 1:**
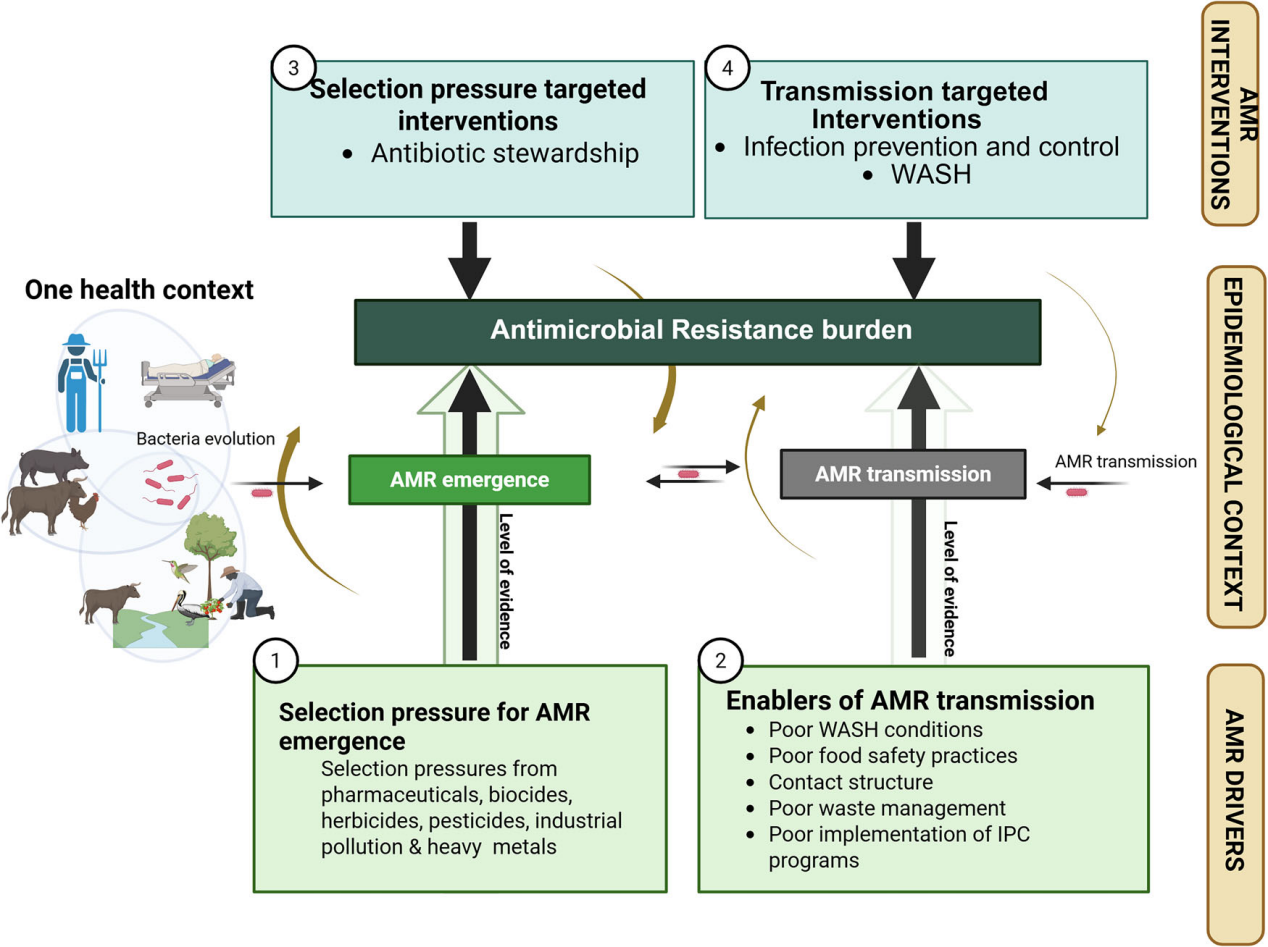
AMR drivers and interventions. Evolution and transmission are the two driving forces for AMR at the interface of human-animal-environment (One health interface). AMR is generated by bacteria evolving, a process that is accelerated by selection pressure (1). Generated AMR is then transmitted across the one health interface with the aid of a range of enablers (2). To effectively combat AMR, interventions must target the two driving forces i.e., (3) Antibiotic Stewardship aimed at limiting the antibiotic use-mediated selection pressure, and (4) Infection prevention and control (IPC). We argue that there is a significant imbalance in the evidence available to inform such interventions, let alone assess their impact. The transparency of the two upward arrows indicates the levels of available evidence i.e., high transparency means low levels of evidence. The brown arrows represent the knowledge-intervention-evaluation cycle existing for each force, and thickness of the arrows indicate the proportional degree. This cycle refers to the knowledge about emergence and transmission of AMR, and how this knowledge is used to inform interventions and evaluate them. The cycle is continuous because the evaluation process creates new knowledge and further inform intervention and so on.

Tackling AMR requires understanding the mechanisms by which resistance to antibiotics emerges, is sustained, and transmitted between bacteria species but also within and between humans, animals, and the environment. Although studying this process has intensified since the launch of the 2015 global action plan on AMR^9^, the holistic understanding of AMR is increasingly complicated due to the significant shifts in demography^10^, the increasing demand on production systems for animal protein production farming^11,12^, and critically, climate emergencies^13^. Additionally, more attention has been directed towards understanding processes associated with emergence of resistant strains and their determinants, as compared to their transmission. Understanding AMR transmission is key for effective control^14,15^. As such, AMR interventions must aim to address both drivers of AMR emergence and AMR transmission in order to effectively manage the AMR burden (Fig. 1).

AMR disproportionately affects low and middle-income countries (LMICs), especially in Africa and Asia^16^. Estimated bacterial AMR associated deaths and attributable deaths were 1.05 million and 250,000 deaths, respectively, in the World Health Organization African region in 2019^4^. Most African countries have only recently established AMR clinical surveillance systems to assess the scale of AMR and are just starting to make inferences about the role of transmission^17^. The scale and characteristics of AMR transmission at the human-livestock interface remain largely unknown due to limited number of studies, most of which are also small and localized. According to the Quadripartite joint secretariat on AMR, understanding AMR transmission at one health interfaces is one of the priority research areas relevant for mobilizing action against AMR in LMICs^18^. Understanding how transmission is defined and determined (inferencing) is a crucial step in supporting the implementation and evaluation of AMR national action plans in Africa. Therefore, this review comprehensively examined how AMR transmission inferences at the human-livestock interface in Africa are made, focusing on (a) how transmission events are defined, (b) the study designs, bacteria species, and data granularity used to support such inferences, (c) frequency of transmission events, and (d) their directional flow between humans and livestock. In doing so, we also evaluate the impact of these factors on the overall strength of the available evidence, forming the foundation for the proposed framework to support robust transmission inferencing.

## Results

### Literature review scope

**Fig. 2:**
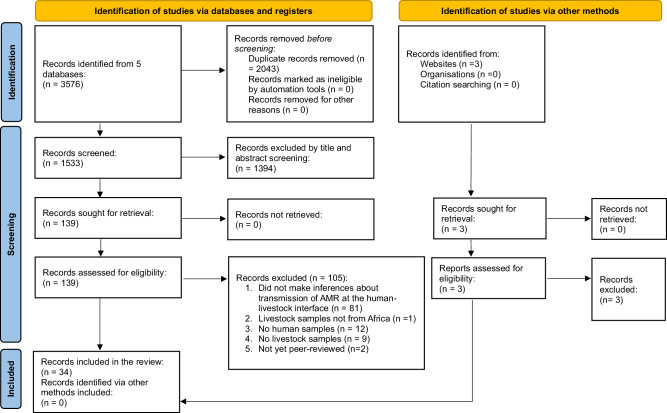
PRISMA flow chart of publication selection. This diagram indicates the systematic process that was followed to include studies captured by our search. We screened 1533 records and included 34 studies in the review.

A total of 3576 records were retrieved from five databases: PubMed (1162), Scopus (824), Web of Science (354), and Ovid Medline and Ovid Embase (1236). The search was from database inception to December 2024. This literature spans 77 years but it was filtered to 34 records (Supplementary Table 2) in the last 11 years as indicated in Fig. 2.

### Temporal and spatial distribution of studies

**Fig. 3:**
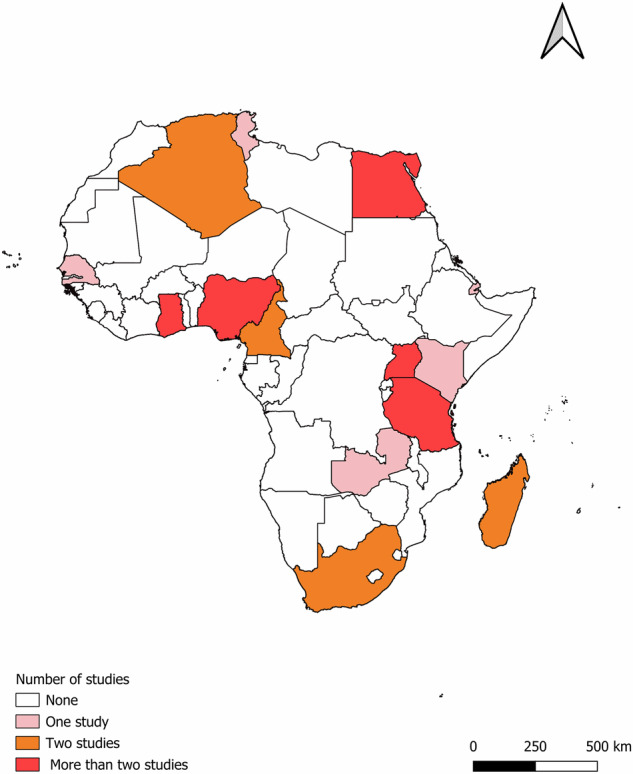
Geographical distribution of reviewed studies (*n* = 34). Countries have been colored based on the number of reported studies included in this review. The map was created using QGIS 3.36.2 (QGIS.org, 2024. QGIS Geographic Information System. QGIS Association. http://www.qgis.org). The shapefile for Africa was downloaded from openAFRICA (https://open.africa/dataset/africa-shapefiles).

The retained studies represent 14 countries across all regions of Africa (Fig. 3), with seven studies from Egypt, four studies each from Uganda and Nigeria, three studies from Tanzania and Ghana, and the remaining nine countries contributing one or two studies each. The included studies were published between 2013 and 2024.

### Study design and quality assessment

None of the studies longitudinally collected samples from study subjects. All but one study^19^ did not report statistical sample size estimations, and only 11 studies incorporated an element of randomization during sampling^19–29^, the majority of studies (23) used non-probabilistic sampling techniques.

While all the studies clearly stated their aims and source populations, only 13 studies explicitly indicated in their study aims that AMR transmission was going to be investigated^21,24,25,27,28,30–37^ (Supplementary Fig. 1). A statement on conflict of interest was explicitly stated in all but two studies^24,38^.

Study quality and strength of evidence was also assessed based on the resolution of bacterial strain typing tools (Supplementary Fig. 1). Sixteen out of 34 studies^20–23,25,27–33,35,37,39,40^ utilized whole genome sequencing (WGS) data, which is currently considered the gold standard due to its high discriminatory power and additional information generated^41,42^. On average, the study design quality of studies was low (Supplementary Fig. 1).

### Biological samples for AMR transmission inferencing

The type of sample from which bacteria are recovered can indicate the potential mode of transmission. In this study, the pooled samples consisted of 22,757 samples, including faecal samples, rectal swabs, hand and nasal swabs, urine, host tissue samples, soil, and water (Supplementary Figs. 2 and 3). These samples were recovered from humans, livestock (cattle, sheep, goats, pigs, chickens), non-conventional livestock species (geese, turkeys, ducks, horses, camels, rabbits), companion animals, wildlife, and the environment (Supplementary Fig. 4). The majority of samples were from livestock (45.81%, *n* = 10,425), followed by humans (35.94%, *n* = 8179) and companion animals and wildlife (12.15%, *n* = 2,764). Only 6.10% of samples were from the environment (*n* = 1389). The median number of samples collected per study was 294 (IQR: 180 - 991), with a range of 65 to 3309 samples. On average, more samples were collected from livestock than humans (Supplementary Fig. 5).

### The bacteria-basis of AMR transmission inferencing

AMR transmission inferences were predominantly based on studies of Gram-negative bacteria (79.41%, 27/34 studies) (Supplementary Table 1). Among these, *E. coli* was most commonly reported (81.48%, 22/27). Other Gram-negative bacteria included *Klebsiella pneumoniae* (5/27), *Salmonella enterica* (4/27), *Klebsiella oxytoca* (2/27), *Acinetobacter baumannii* (1/27), *Proteus mirabilis* (1/27), *Serratia fonticola* (1/27), *Kluyvera ascorbata* (1/27), *Enterobacter cloacae* (2/27), *Enterobacter aerogenes* (1/27), *Raoultella ornithinolytica* (1/27), *Cronobacter malonaticus* (1/27), *Citrobacter werkmanii* (1/27), and *Pluralibacter gergoviae* (1/27). In contrast, seven out of the thirty-four studies (20.59%) examined Gram-positive bacteria with *Staphylococcus aureus* being the most frequent (5/7) (Supplementary Table 1). Other reported Gram-positive species included *Enterococcus spp*. (1/7) and coagulase-negative staphylococci (3/7).

### The resolution of tools defining the unit of AMR transmission

Here the resolution of tools was categorized into (i) very low i.e., antibiotic resistance (AMR) phenotyping, (ii) low i.e,. PCR methods, sequencing of AMR gene amplicons and multi-locus sequence typing (MLST), (iii) intermediate i.e,. pulse-field gel electrophoresis (PFGE), and iv) high-resolution for the use of whole genome sequencing (WGS). Forty-seven percent of the studies used high-resolution tools to examine bacterial strains and their resistance characteristics, while 53% used intermediate, low or very low-resolution tools. The shift towards WGS has accelerated in the recent years. In the last 5 years, 60% of the included studies used WGS.

### Powering studies that inform AMR transmission inferencing

**Fig. 4:**
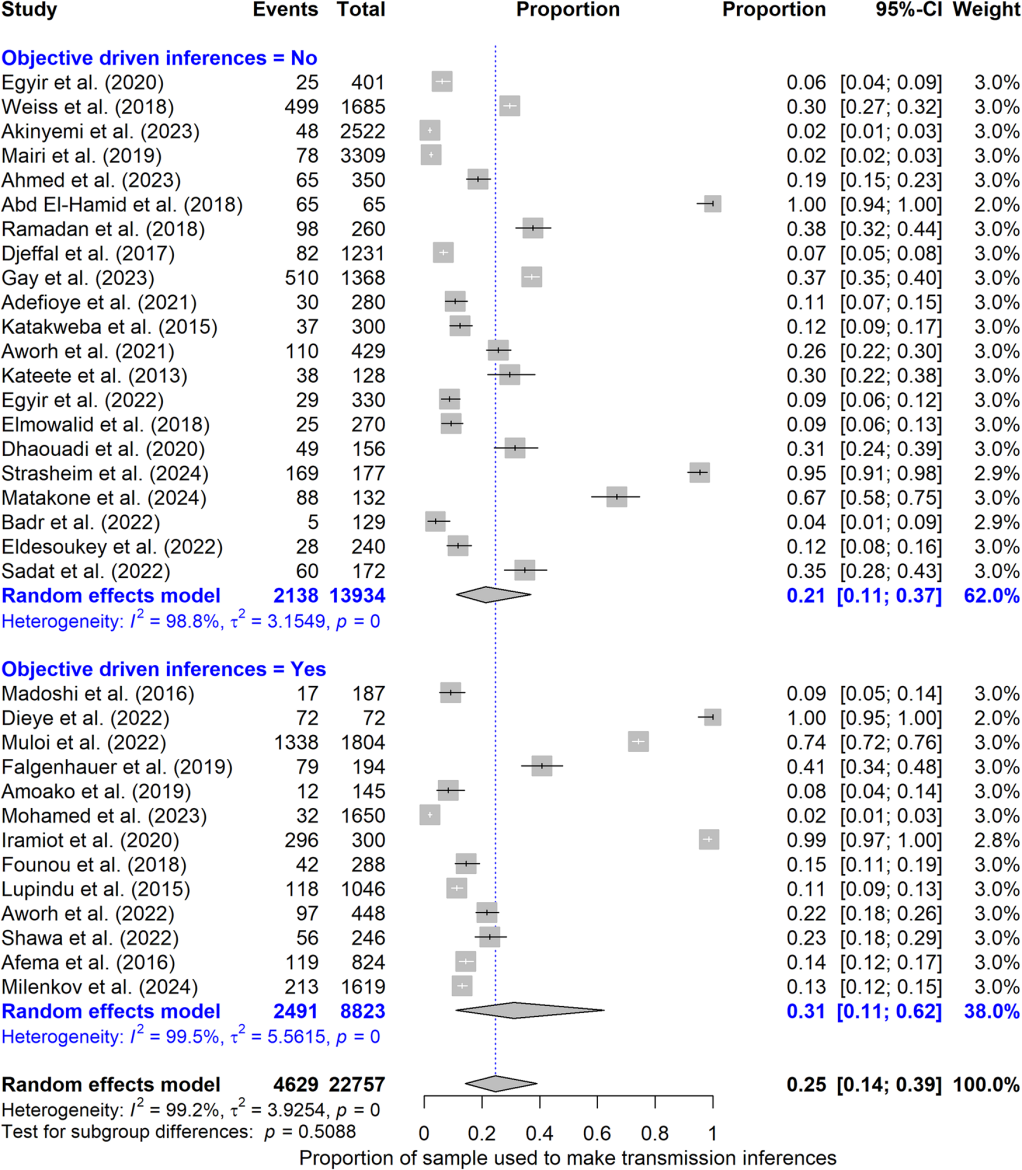
Proportion of the sample size that was used to make antibiotic resistance transmission inferences in 34 studies. The X-axis is the proportion for individual studies as listed along the Y-axis, with the range of proportion in 95% confidence interval (CI). The position and size of the boxes represent proportion and weight of the study in the meta-analysis, respectively. The pooled proportion is represented by the diamond. Events, which is the numerator, refer to the number of samples used for investigation of transmission in a study, while Total, the denominator, refers to the total number of samples collected in a study. The objective driven inferences (Yes/No) variable categorizes studies into whether they explicitly stated in their study aims that they were going to investigate transmission or not.

We note that not all collected samples were used for AMR transmission inferencing. The pooled size of the sample used for transmission inferencing was 4629, representing 35.26% from humans, 46.99% from livestock, and 17.76% from companion animals, wildlife and the environment. The pooled proportion of samples used for AMR transmission inferencing in the 34 studies as estimated by the meta-analytic method was 25% (95% CI: 14–0.39%, *I*^*2*^ = 99.2%) (Fig. 4) (Supplementary Fig. 8). The proportion of samples used for AMR transmission inferencing across studies was heterogeneous (Conchran’s *Q*-test, *P* < 0.05). Descriptively, the proportion of collected samples used for investigating transmission ranged from 1.90 to 100%, with a median of 16.58% (IQR: 9.13–36.68%). This proportion was substantially variable across the studies, as supported by the high levels of heterogeneity in the meta-analysis, reflecting differences in bacteria studied, study aims, spatial and temporal setting, and laboratory capacity among other factors. The median sample size used for inferencing in a study was 65 (IQR: 33–107). On average, livestock samples were significantly more frequent than human samples in AMR transmission inferencing (Wilcoxon signed rank test, *P* < 0.05) (Supplementary Fig. 6). Although not statistically significant, it is noteworthy that studies that explicitly stated in their study aims that they were going to investigate transmission used more samples to infer transmission than those that did not.

### Defining AMR transmission events at the human-livestock interface

Various keywords were used in transmission inferences with “transmission” being the most frequent (74%) (Supplementary Fig. 7). Other keywords included circulation, diffusion, dissemination, exchange, sharing, spread and transfer. Of the 34 studies, 30 (88.24%) concluded that there was transmission of antibiotic-resistant bacteria or their resistance determinants between humans and livestock, while the remaining studies inferred no transmission. Among the studies that concluded occurrence of transmission, one^34^ based its inference on the co-occurrence of AMR phenotypes whereas half of the studies (15/30, 50%) used WGS to investigate transmission. In most of WGS-based studies, genetic relatedness was assessed using whole genome or core genome single-nucleotide polymorphism (SNP) or core genome MLST distances. However, one WGS-based study^40^ used the traditional 7-gene MLST scheme to evaluate genetic similarity. For the remaining studies, transmission inferences were based on similarity of PFGE band patterns in three studies, PCR methods in five studies, traditional MLST in four studies, and AMR gene sequences in one study. On the other hand, among the studies concluding that transmission did not occur, only one used WGS^20^. The other three relied on PFGE^43^, traditional MLST^44^, and PCR methods^45^. Regarding the unit of inference, the basis of most studies (21/30) was transmission of antibiotic-resistant bacteria, while the remaining were based on resistance determinants or both.

### Defining direction of AMR transmission at the human-livestock interface

Out of 30 studies that inferred transmission, only ten^30–32,40,46–51^ inferred the direction of that transmission. Of the ten studies, seven did not specify the basis on which inferences about the direction of transmission were made. For the three that did, one study inferred a human-to-goat transmission based on the presence of human-adapted virulence genes (*scn* and *sak*) in *S. aureus* isolates recovered from a goat^40^. The second study inferred a human to chicken transmission based on the presence of a human-linked MRSA clone, MDR-MRSA clone ST612-CC8-t1257-SCCmec_IVd (2B), in poultry^32^. The third study suggested bidirectional transmission of MRSA due to genetic similarity between livestock-associated and community associated MRSA^47^.

### Quantification of AMR transmission

**Fig. 5:**
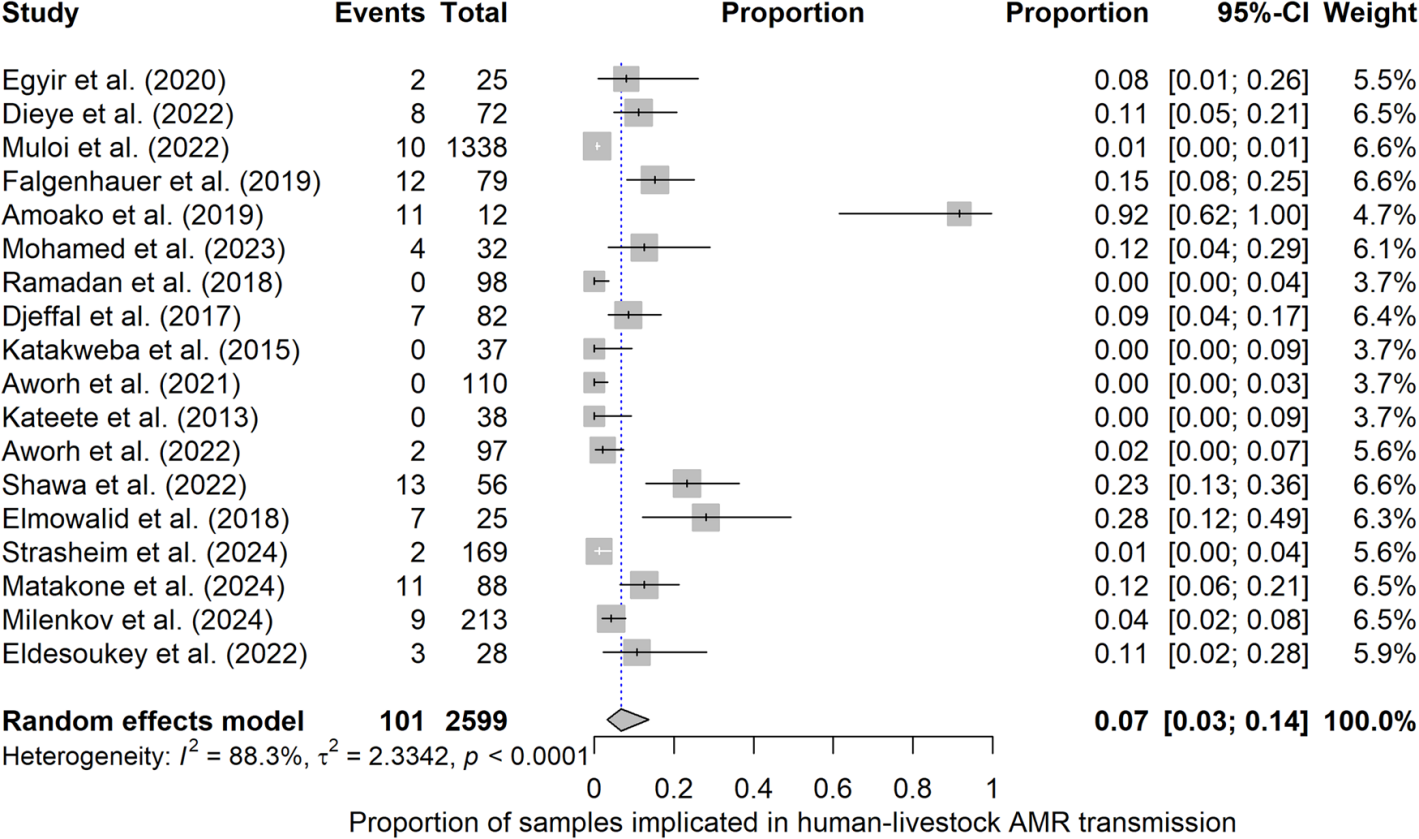
A pooled estimate of the proportion of individuals involved in transmission events at the human-livestock interface based on 18 studies. The X-axis is the proportion for individual studies as listed along the Y-axis, with the range of proportion in 95% confidence interval (CI). The position and size of the boxes represent proportion and weight of the study in the meta-analysis, respectively. The pooled proportion is represented by the diamond. Events, which is the numerator, refer to the number of individuals implicated in human-livestock AMR transmission, while Total, the denominator, refer to the total numbers of individuals used for investigation of transmission in a study.

No study presented quantitative evidence on the frequency of AMR transmission events. Information on statistical probability associated with inferred transmission events was also unavailable in all the studies. Despite this limitation, a pooled estimate of individuals implicated in human-livestock AMR transmission was estimated by quantifying the proportion of hosts explicitly reported to be implicated in human-livestock AMR transmission in 18 studies (Fig. 5). It is noteworthy that four studies reported no transmission, therefore they were implicitly assigned zero individuals. The estimated proportion of individuals involved was 7% (95% CI: 3–14%) (Fig. 5) (Supplementary Fig. 9).

## Discussion

In this review, we comprehensively examine the current literature on antibiotic resistance transmission at the human-livestock interface in Africa, focusing on how transmission is defined, how studies are designed to retrospectively identify transmission events, the bacteria involved, the resolution of data used to support transmission inferences, the frequency of transmission events as well as their direction at the human-livestock interface. This approach uniquely allows us to assess the current knowledge about transmission and the quality of existing evidence. Our motivation is to find solutions to the critical challenge that transmission poses to current AMR control strategies. Our review shows that the strength of evidence has improved significantly in recent years due to increasing adoption of high-resolution WGS for typing pathogens. However, the imprecise definition of the transmission process, limited population representativeness due to use of non-probabilistic sampling approaches and lack of suitable and defined methods to power such studies, lack of metrics to quantitatively describe uncertainty and summarize information about transmission, and a focus on a few high-risk pathogens (limited pathogen scope) continue to devalue the strength of evidence and potentially limits our understanding of AMR transmission.

### Imprecise AMR transmission definitions undermine evidence quality

A clear definition of a process lays the foundation for standardized and reproducible protocols used by an active community of practice. This review highlights variation in how transmission events are defined, mainly due to differences in the resolution of tools used to determine the relatedness of bacterial strains recovered from individuals involved in a transmission event. Broadly, transmission was defined based on genetic relatedness and spatiotemporal connectedness of isolates involved. Some studies used similarity of resistance phenotypes instead of genetic relatedness. However, we recognize that the molecular characteristics driving such phenotypes can vary across bacteria^52^, therefore weakening the strength of inferences made from such evidence. Even with molecular data, the definition of genetic relatedness varied across strain typing methods. Within strain typing methods, thresholds for defining genetic similarity were also various. Furthermore, the degree to which epidemiological contextual data was incorporated when making AMR transmission inferences was highly variable across the studies. All such variations could be used for characterization of the quality of available AMR transmission evidence. We also recognize that it is challenging to prescribe a single definition for an AMR transmission event that is applicable to all species of bacteria. This is because molecular characteristics, population structure and spatial and temporal epidemiology characteristics are different across species of bacteria. Nevertheless, a standardized definition of AMR transmission, whether at the species or strain level, would improve the definition of a transmission event and enable meaningful comparisons while informing targeted control strategies across clinically important bacterial pathogens.

### Inference strength relies on study design and molecular resolution

The representativeness and statistical confidence associated with estimates are integral to the strength of evidence^53,54^. Here we acknowledge the complexity behind making inferences of AMR transmission, particularly at the human-livestock interface. The literature shows that this process involves identifying the optimal samples likely to reveal transmission events, selecting the appropriate anatomical samples, isolating a pathogen or commensal that retains a stable signal of the underlying AMR transmission network, and using the right tools to decipher it. This means that any laxity at any of these processes weakens the strength of the inferences drawn. There is a need to establish experimentally informed thresholds of genetic similarity at which transmission can be inferred. This review shows that the strength of evidence is often compromised by the lack of robust study powering and use of cross-sectional study designs, and use of low-resolution typing methods. This is particularly because transmission is a dynamic process that is retrospectively studied and that strong transmission inferences rely on high-resolution representative posterior evidence. Here, WGS and phenotype co-occurrence based inferences carry the highest and least confidence, respectively. The former method allows for both allelic level and SNP distance relatedness assessment. Beyond longitudinally sampled genomic data, high-quality contextual metadata is essential for refining the temporal, spatial, and host characteristics that underpin a transmission event.

### AMR transmission inferencing in Africa is primarily based on *Escherichia coli*

*E. coli* accounts for 65% of the pathogens used for AMR transmission inferencing in Africa. However, its “promiscuity” as a highly diverse species with a broad host range and a large accessory genome^55^ can complicate transmission inferencing. This is especially relevant in the absence of a refined genomic analysis plan or comprehensive supporting epidemiological context. An approach that uses multiple species of bacteria, including strain level clonal complexes, is likely to yield better insight into transmission dynamics, particularly at the human-livestock interface. The depth of such insights can further be improved by using temporal-rich data generated from longitudinal designs. Although *E. coli* accounts for 1 in 5 global foodborne infections^56^, predominantly of animal origin, its dominance in AMR inferencing likely represents only a partial view of the true nature of transmission.

### AMR transmission studies are underpowered

The proportion of samples used for AMR transmission inferencing, relative to the total number of samples collected, was substantially low. This is likely due to insufficient resources for comprehensive phenotypic and genotypic bacterial characterization and the inability to isolate target bacteria, stemming from limited laboratory capacity, suboptimal sampling sites, bacterial and AMR prevalence, and sample types. Consequently, significant power to uncover AMR transmission networks is lost due to the reduced number of samples used in downstream analyses. There is also a lack of suitable methodologies to determine optimal sample sizes for robustly powering transmission studies. For instance, the only study^19^ that calculated sample size used the Lorentz formula: ${\rm{n}}=\frac{p\left(1-p\right){z}^{2}}{{d}^{2}}$, where (p) represents the prevalence of AMR bacteria of interest, (z) is the Z-score for the desired confidence level, and (d) is the margin of error. However, in this study, the calculated sample size (n) applied to the estimation of the proportion of pigs expected to carry resistant bacteria of interest rather than estimation of the frequency of transmission of resistant bacteria of interest, as (p) reflects the proportion of resistant bacteria, not the proportion of transmission events. In addition, Fig. 4 shows no statistical difference in pooled estimate of sample size used for inferencing between studies explicitly investigating transmission and those that did not, suggesting that study underpowering arises from far more complex factors including experimental costs and downstream design limitations.

### Quantitative AMR transmission inferencing remains a gap

Effective infectious disease control requires examining both scale and spatial distribution to inform targeted interventions^57,58^. The same holds true for AMR, but the review highlights challenges in methodological approaches for estimating transmission frequency which may explain the paucity of evidence. Although the pooled estimate of individual hosts implicated in human-livestock AMR transmission was 7%, this likely underrepresents the true extent of transmission due to inherent limitations in study design. Addressing these gaps should be a priority for future research. Regardless of the existence of biologically plausible hypotheses about transmission of AMR at the human-livestock interface, incorporation of differences in the strength of evidence to characterize the strength of transmission inferences is often neglected. At best, current studies tend to use a binary response—presence or absence of transmission—without attaching any measure of confidence, thereby overlooking the uncertainties inherent in such retrospectively inferred processes. To improve the applicability of inferences made, it is important to formally attach to our inferences the level of confidence in our beliefs about specific transmission events considering that these beliefs might have various degrees of confidence. Such can be achieved through development of probabilistic frameworks that rely on integration of epidemiological contextual data and molecular data to infer transmission. Furthermore, estimates of transmission frequency could aid sample size calculations.

Studies outside Africa similarly reveal that research on AMR transmission often relies on cross-sectional designs, with limited attention to formal sample size calculations, and scarce quantitative transmission data^59–64^. This common methodological gap underscores the need for standardized frameworks to guide study design, sample size determination, and quantification of frequency and probability associated with transmission events.

### A proposed framework to support AMR transmission inferencing

Reflecting on the existing evidence and current knowledge of AMR transmission, we propose a framework (Table 1) which could inform AMR transmission studies and improve resulting inferences. The framework presents the AMR transmission inferencing pathway in 5 key logical steps and their associated contexts for intervention (included in the brackets) as follows: (1) Defining AMR epidemiological context (the foundation for implementation and evaluation); (2) Isolating bacteria (AMR route of transmission and potential route for intervention); (3) AMR phenotyping (basis for clinical intervention); (4) AMR genotyping (defining the unit of transmission); and (5) AMR transmission inferencing (source of evidence to trigger intervention). In addition, the framework also highlights gaps, makes recommendations, and indicates some opportunities that exist on each of the steps along the transmission inferencing pathway.

**Table 1:** AMR transmission inferencing framework

Transmission inference pathway	Gaps	Recommendation	Future opportunities
1. Defining epidemiological context **Critical considerations** - Selecting a relevant geographical context (Density-bases i.e., urban vs rural) - Define transmission - Document assumptions on; a) level of host interaction, b) routes of transmission, therefore the relevant sample type - What is the likelihood of recovering the sentinel pathogen from such a sample? - Then define the required depth of sampling (Cross sectional Versus Longitudinal) - If data is required via surveillance or clinical investigation the consideration above will vary - Finally, use the above information to determine the appropriate sample size	- Lack of standardized methodological framework for determining optimal sample size for transmission inferencing - Low study powering (Breadth) - Lack of depth (Longitudinal) - Limited representativeness(Scope) - Limited diversity (Host-host)	- Develop methodology for sample size estimation, where the prevalence/frequency of transmission event is the statistic - Longitudinal studies to add depth and maximize detection of transmission networks - Probabilistic sampling to improve representativeness - Or Risk based sampling to maximize detection	- A transmission inference framework will enable the use of large archived genome and metadata datasets for retrospective transmission analysis - Real-time transmission network mapping can enhance sample size estimation - Cross species transmission studies
2 Isolating bacteria **Critical consideration** To select a pathogen, consider clinical priority^69^ and the likelihood of recovery given a sample and route - Public health surveillance: sentinel bacteria (high probability of recovery) - Clinical surveillance: pathogenic bacteria(Clinical priority) - Standard microbiology- It is critical to use standard methodology to allow comparability - Bacteria and sample type infers route of transmission	- *E. coli* dominates transmission inferences limiting insights - Predominantly culturable microbes investigated	- Use more pathogens to study transmission to broaden insights, i.e., Gram + and – concurrently for relevant transmission routes - Use clonal pathogens/strains to support development of methodology^a^ - Use of microbial populations for inferencing	- Adopting routine metagenomics can improve transmission inferencing by including signals across microbial populations, revealing co-occurrences and resistance patterns
3 AMR Phenotyping **Critical quality control processes** - Selection of antibiotics of interest depending on 1& 2 (Antibiotics choice is informed by clinical management guidelines) - Disc diffusion versus MIC	- Low resolution - Mainly Disc diffusion used (Error in reading)	- Improve resolution and accuracy by using MIC^70–73^ - Define the purpose of the phenotype, i.e., clinical or public health investigation	- With 90% concordance between AMR phenotype and genotype, rapid diagnostic readouts can enhance their utility for transmission tracking, particularly in hospitals
4 AMR Genotyping **Critical considerations** - DNA extraction methods and quality control - Contamination minimization - Granularity maximization - Tools for clustering relatedness	- Low-resolution tools - Lack of Standardization - Underutilization of molecular output	- Use whole genome sequencing data for strain typing - Complement short-read whole genome sequencing data with long reads to study the context of AMR genes and plasmids associated with transmission	- Improving the granularity of PCR-based tools, including low-cost multiplexing of phenotype and genotype, enables LMICs to trace AMR effectively and generate datasets for transmission inferencing
5 AMR transmission inferencing **Critical considerations** - Contextualizing the definition of transmission - Determining genetic relatedness threshold for defining transmission	- Lack of evidence to support study designs especially power and sample sizes - Lack of quantification - Uncertainty unaccounted for - Lack of robust metadata - Lack of directionality	- Integrate high-quality epidemiological data with high-resolution genomic data - Develop quantitative probabilistic frameworks for inferring transmission - Develop methods for quantifying the magnitude of AMR transmission in populations	- Utilization of next-generation and third-generation sequencing technologies, and artificial intelligence applications to study transmission of AMR and infer directionality

Table describes the transmission inferencing pathway and critical considerations for each step, identified gaps, recommendations and opportunities.

^a^Clonal bacteria generally maintain stable genetic lineages and could play an important role in studies aimed at developing genetic distance thresholds for defining transmission events in specific bacteria species or strains. The “critical considerations” are analogous to critical control points - specific stages in the AMR transmission inference pathways where care ought to be taken to improve the quality of downstream inferences.

#### Recommendations for Prospective Researchers

Building on the recent improvements in the strength of inferences documented here, we recommend the following:

a) *Strengthening epidemiological study designs*: Focus on enhancing representativeness by using probabilistic sampling to reduce random errors and minimize bias. Additionally, ensuring adequate study power will increase the likelihood of detecting transmission events. We highlight key differences between conventional sample size estimation based on a priori prevalence and that required for transmission studies. In prevalence studies, the unit of estimation is the individual, while for transmission, it is the pair involved in the transmission event. This distinction may violate conventional principles of sample size estimation, underscoring the need for future research to refine the framework for sample size calculation for transmission studies.

b) *Expanding the range of bacteria species*: We recommend incorporating a broader range of bacteria species to better elucidate the transmission landscape of AMR at the human-animal interface.

c) *Using high-resolution sequence data:* Integration of high-resolution sequence data with good epidemiological narratives is crucial for unraveling AMR transmission networks. However, due to high cost of WGS, it is important to compare WGS (as a gold standard) and low-resolution tools to identify a combination of the later which can be used with greater resolution or inform the development of rapid and low-cost assays with adequate resolution to support targeted AMR surveillance and transmission studies in LMICs.

### Recommendation for policymakers and funders

Given the critical role that transmission plays in AMR, there is a dire need to invest in generating and promoting the use of robust evidence to tailor interventions and mitigate its transmission in Africa.

*In conclusion*, the growing use of granular tools to unravel AMR transmission events has improved the quality and strength of inferences. However, upstream components, especially study designs, need to be strengthened to further bolster the evidence that informs policy and the implementation of effective AMR control strategies.

## Methods

This systematic review was conducted in accordance to Preferred Reporting Items for Systematic Reviews and Meta analyses (PRISMA) standards^65^ (Supplementary Table 3). The protocol for conducting this systematic review was registered with the International Prospective Register of Systematic Reviews (PROSPERO) (https://www.crd.york.ac.uk/prospero/) under the identification number CRD42024589051.

### Literature search

Peer-reviewed literature was systematically searched in the following electronic databases: PubMed, Scopus, Web of Science, Ovid Medline and Ovid Embase. A search strategy tailored to each of the five electronic databases was developed and included the following search terms: (“Antimicrobial resistan*“ OR AMR OR ABR OR “Antibiotic resistan*“ OR “Drug resistan*“ OR “Antibacterial resistan*“ OR “Multi-drug resistan*“ OR “Extensively drug resistan*“ OR “Bacterial resistance”) AND (Bacteria OR Bacteri*) AND (Transmission OR Spread OR Transmi* OR spread* OR Transfer* OR Exchang* OR Shar*) AND (human* OR people OR person*) AND (Livestock OR Animal* OR Pig OR swine OR Cattle* OR Goat* OR Sheep OR Ruminant* OR Poultry OR Chicken*) AND (Africa OR “Sub-Saharan Africa” OR “East Africa” OR “West Africa” OR “North africa” OR “Southern Africa” OR Africa* OR Algeria OR Angola OR Benin OR Botswana OR “Burkina Faso” OR Burundi OR “Cabo Verde” OR “Cape Verde” OR Cameroon OR “Central African Republic” OR Chad OR Comoros OR Congo OR “Democratic Republic of the Congo” OR “Republic of the Congo” OR “Côte d’Ivoire” OR “Ivory Coast” OR Djibouti OR Egypt OR “Equatorial Guinea” OR Eritrea OR Eswatini OR Swaziland OR Ethiopia OR Gabon OR Gambia OR Ghana OR Guinea OR “Guinea-Bissau” OR Kenya OR Lesotho OR Liberia OR Libya OR Madagascar OR Malawi OR Mali OR Mauritania OR Mauritius OR Morocco OR Mozambique OR Namibia OR Niger OR Nigeria OR Rwanda OR “São Tomé and Príncipe” OR Senegal OR Seychelles OR “Sierra Leone” OR Somalia OR “South Africa” OR “South Sudan” OR Sudan OR Tanzania OR Togo OR Tunisia OR Uganda OR Zambia OR Zimbabwe). All searches spanned from database inception to December 2024 and included original research articles published in English language. We also searched the World Health Organization, Food and Agriculture Organization, and World Organization for Animal Health websites for information relating to transmission of antibiotic resistance.

### Inclusion criteria

Criteria used for screening and selection of records were based on PRISMA guidelines^65^ as illustrated in Fig. 2. The search mainly focused on mapping existing literature on antibiotic resistance transmission inferences at the human-livestock inferences in Africa. Covidence (https://www.covidence.org/) was used to remove duplicates from a pool of 3576 records that was generated from the 5 databases. After removing duplicates, title and abstract screening was conducted for the remaining 1533 records, where records not focusing on antibiotic resistance transmission between humans and livestock were removed. Screening was independently done by FC and validated by AM. The resulting 139 records were then checked for meeting the inclusion criteria. A record was included if it was published in English language, conducted in Africa, made inferences about transmission of antibiotic-resistant bacteria and/or their resistance determinants (mobile genetic elements and antibiotic resistance genes) at the human-livestock interface, included bacterial isolates from both humans and livestock, and was original research.

### Data extraction

Data was extracted from 34 records that fully met the inclusion criteria. Data were first extracted by FC and independently checked by AM using a checklist which was initially agreed upon by authors. Data were extracted and entered in an MS Excel spreadsheet with multiple variables including authors, year of publication, study design, bacteria pathogens studied, typing methods, sampling units, sample size, conclusions about transmission, and directionality of transmission.

### Quality assessment

To assess a study’s methodological quality, we framed a simple checklist adapted from a tool based on the Strengthening the Reporting of Observational Studies (STROBE) statement^66^. This assessed selection of study subjects, measurement of variables for making transmission inferences, potential biases and measures used to control biases, power and sample size calculation, and conflicts of interest. A study was considered to have used a valid and reliable variable measurement instrument if they employed WGS to generate data for AMR transmission inferencing.

### Data analysis

Using the R Meta package^67^, a random effects inverse variance meta-analysis of proportions with logit transformation was performed in R version 4.3.2^68^ to (a) calculate the average proportion of the samples in a study that were used to make inferences about transmission of antibiotic resistance between humans and livestock and (b) estimate the proportion of individual hosts implicated in human-livestock AMR transmission. A continuity correction of 0.5 was applied to studies with proportions equal to 0 or 1 to avoid computational errors in the transformation. The DerSimonian-Laird estimator was used for between-study heterogeneity. Forest plots were used to visualize individual and pooled estimates with 95% confidence intervals. Funnel plots were used to assess publication bias, with asymmetry evaluated visually. Due to the non-normality of the data as assessed by the Shapiro-Wilk test, Wilcoxon signed-rank test was also performed in R to test whether human and livestock sample sizes significantly differed in a study. QGIS 3.36.2 was used to generate the map indicating the geographical distribution of studies included in this review.

## Supplementary information

Supplementary information

## Data Availability

Extracted data and code are available on OSF at www.fmdbase.org.
