# Supplementary material for: A systematic review of antimicrobial resistance transmission inferences at the human-livestock interface in Africa
Source: NPJ Antimicrob Resist. 2025 Jun 30;3:58. doi: 10.1038/s44259-025-00126-y (PMC12209416; doi:10.1038/s44259-025-00126-y)
Supplement: Supplementary file 1 — Supplementary information [file 44259_2025_126_MOESM1_ESM.pdf]

Supplementary information for

A systematic review of antimicrobial resistance transmission inferences at the human-livestock interface in Africa

Frank Chilanga, Kenneth Kasozi, Stella Mazeri, Gavin K. Paterson, Adrian Muwonge

Content:

Supplementary Figure 1 - Quality assessment of included studies

Supplementary Figure 2 - Types of human samples reported in the reviewed studies

supplementary Figure 3 - Types of livestock samples reported in the reviewed studies

Supplementary Figure 4 - Sources of samples in the 31 studies included in the review

Supplementary Figure 5 - Human and livestock sampled in a study

Supplementary Figure 6 - Human and livestock samples used for AMR transmission inferencing at the human-livestock interface

Supplementary Figure 7 - Relative frequency of keywords used in transmission inferences

Supplementary Figure 8 - Funnel plot of standard error vs. logit-transformed proportions for 34 included studies assessing the proportion of collected samples used for AMR transmission inferencing

Supplementary Figure 9 - Funnel plot of standard error vs. logit-transformed proportions for 18 included studies assessing the proportion of individuals involved in AMR transmission at the human-livestock interface.

Supplementary Table 1 - Species of bacteria reported in included studies

Supplementary Table 2 - Summary of included studies

Supplementary Table 3 - PRISMA 2020 Main Checklist

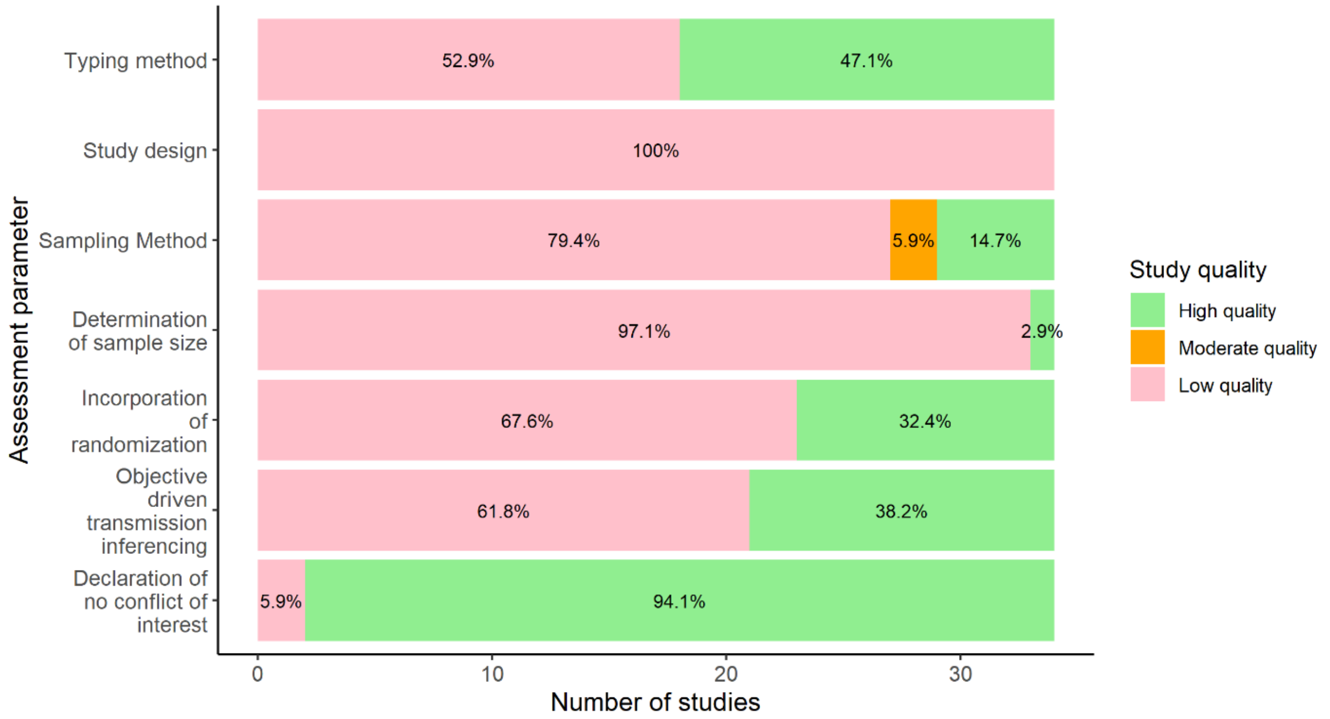

Supplementary Figure 1: Quality assessment of 34 included studies**.**

1. **Typing methods**: A study was considered to be of high quality when WGS was used and low quality if otherwise.
2. **Study design**: A study was considered to be of high quality when a longitudinal design was implemented, and low quality if otherwise.
3. **Sampling method:** A high-quality study employed random sampling, a moderate quality study combined judgemental, convenience or systematic sampling with random sampling, a low-quality study solely used non-probabilistic sampling approaches.
4. **Determination of sample size:** A study was considered high quality if sample size was calculated prior to implementation of the study, otherwise it was considered to be of low quality.
5. **Incorporation of randomization**: Quality was considered high if an element of randomization was incorporated in the study, otherwise, the quality was low.
6. **Objective driven transmission inferencing**: The quality of a study was considered high if it was explicitly stated in the study's objectives that AMR transmission was going to be studied. If otherwise, the quality was considered low.
7. **Declaration of no conflict of interest:** Quality was high if it was declared that all the authors had no conflict of interest, otherwise, the quality was low.

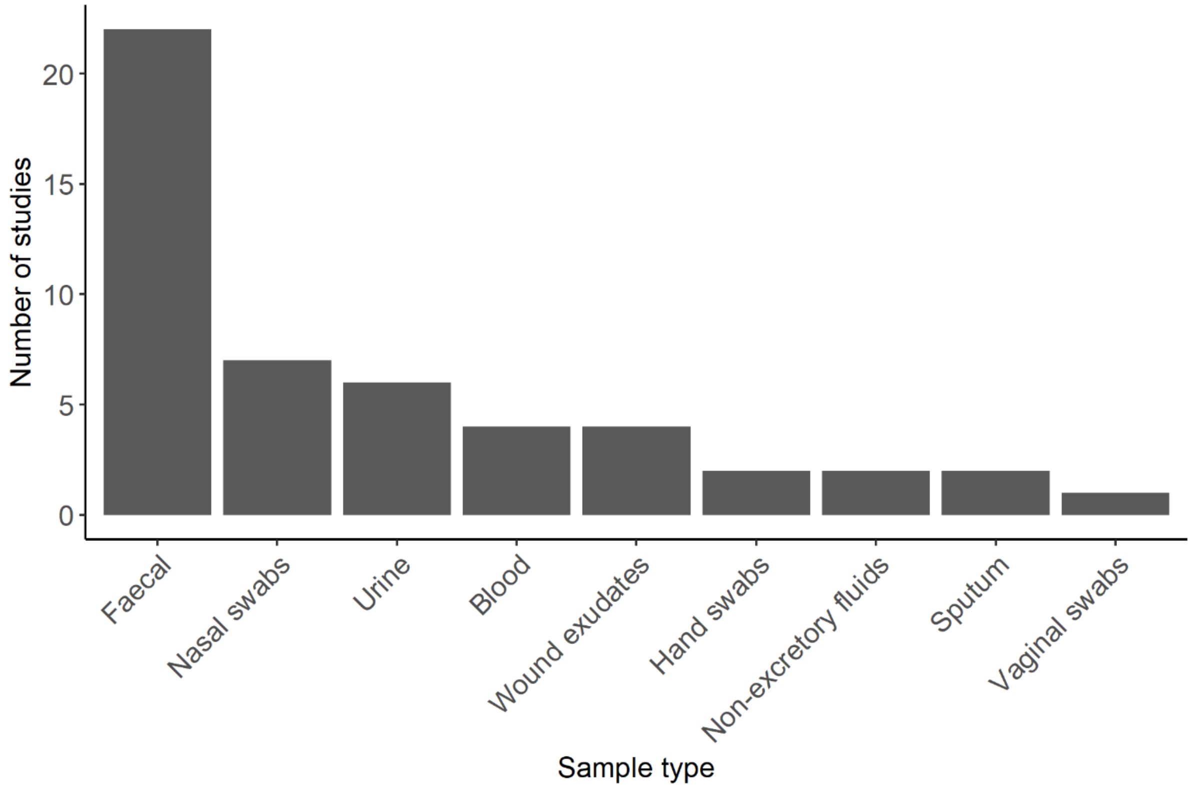

Supplementary Figure 2: Types of human samples reported in the reviewed studies. Non-excretory body fluids included cerebrospinal and pericardial fluids.

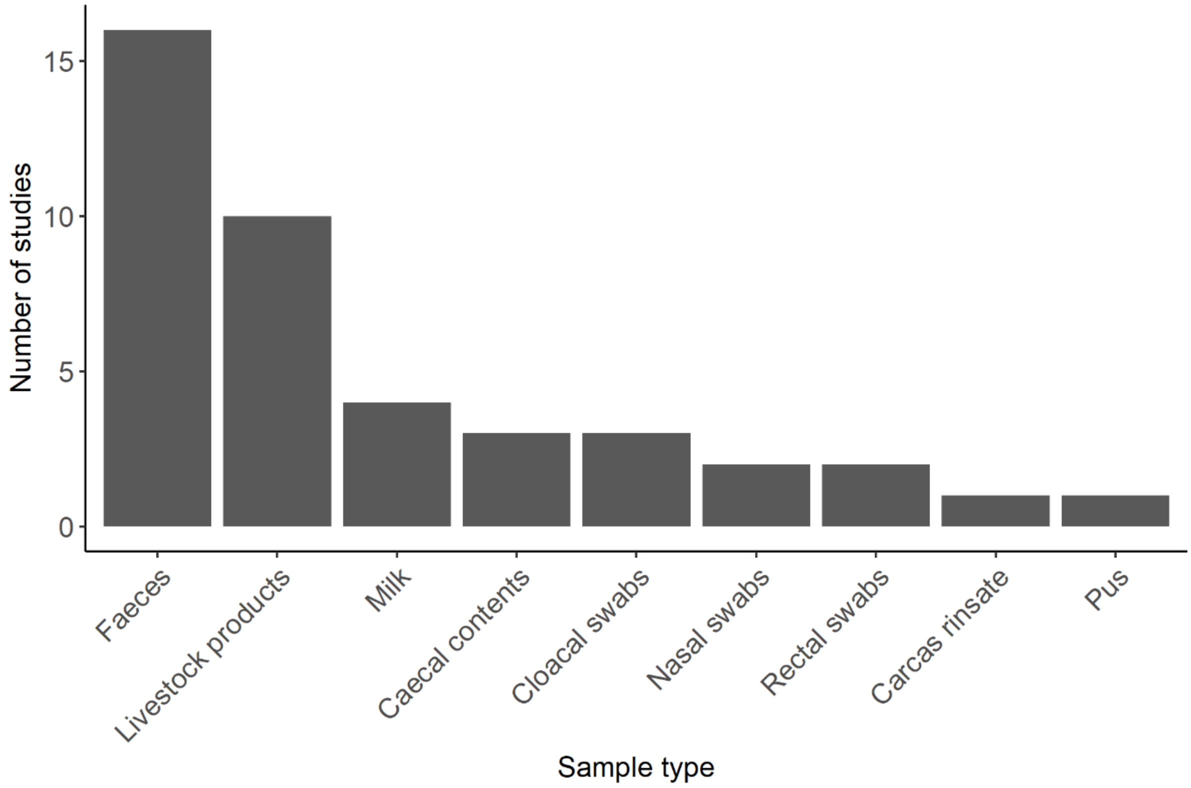

Supplementary Figure 3: Types of livestock samples reported in the included studies.

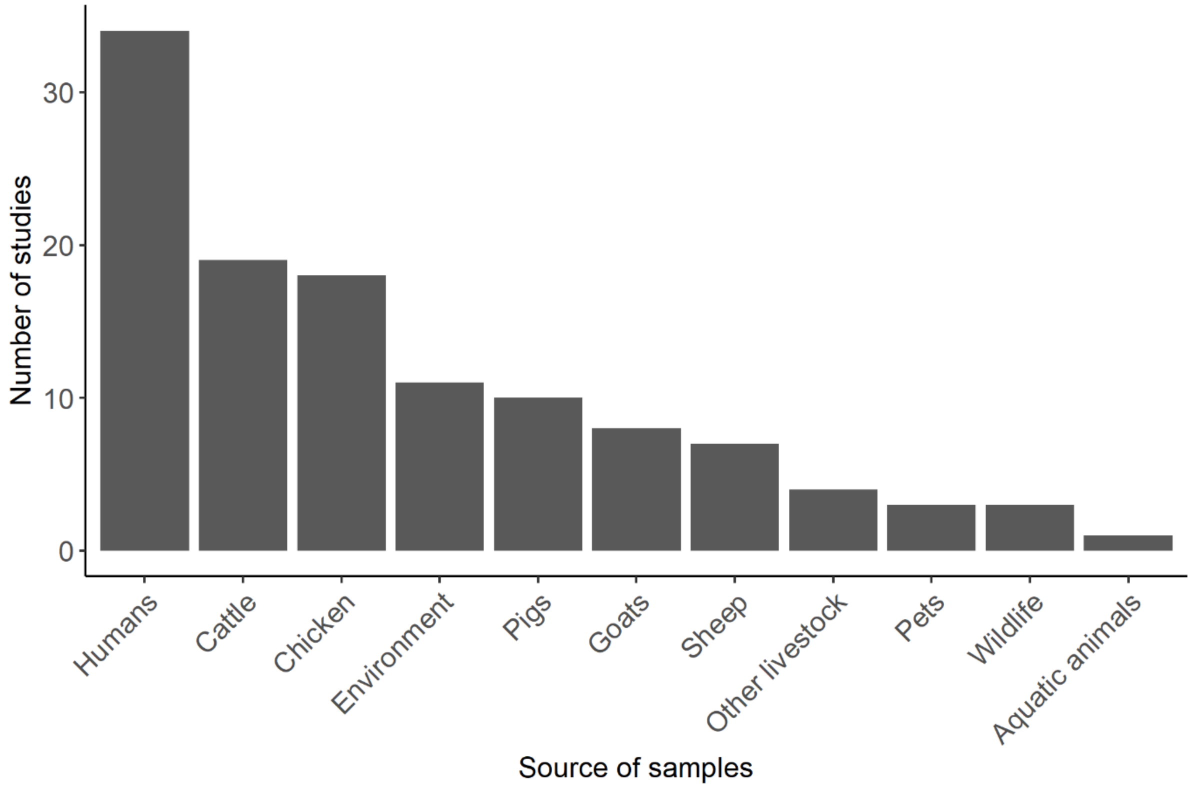

Supplementary Figure 4: Sources of samples in the 34 studies included in the review. Other livestock included geese, turkeys, ducks, horses, camels, and rabbits.

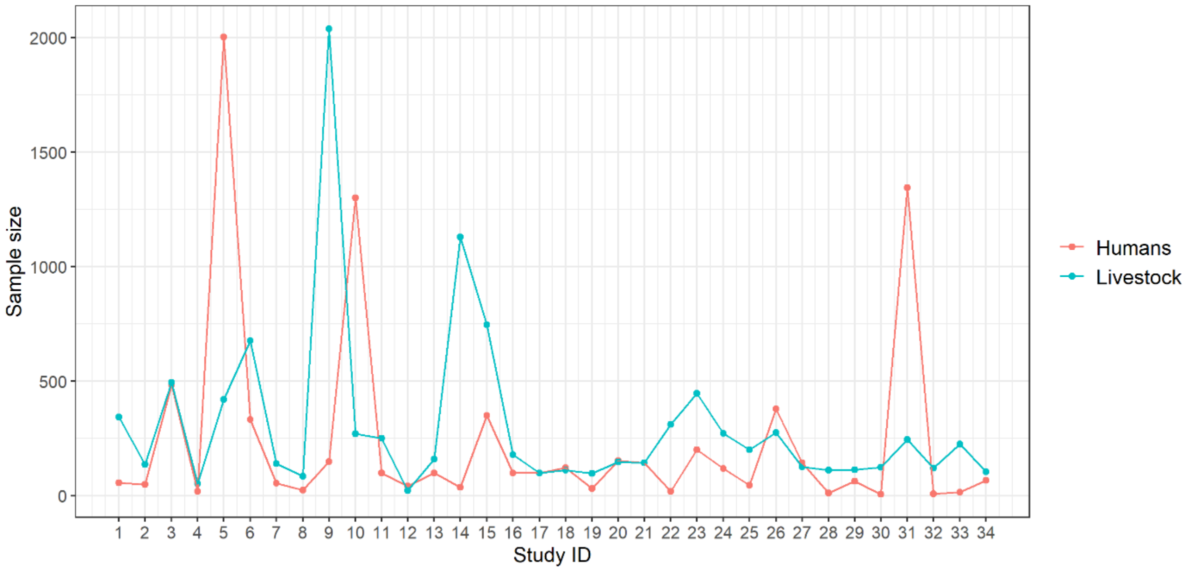

Supplementary Figure 5: Human and livestock samples collected in a study. The x axis indicates individual study with its respective human and livestock sample size.

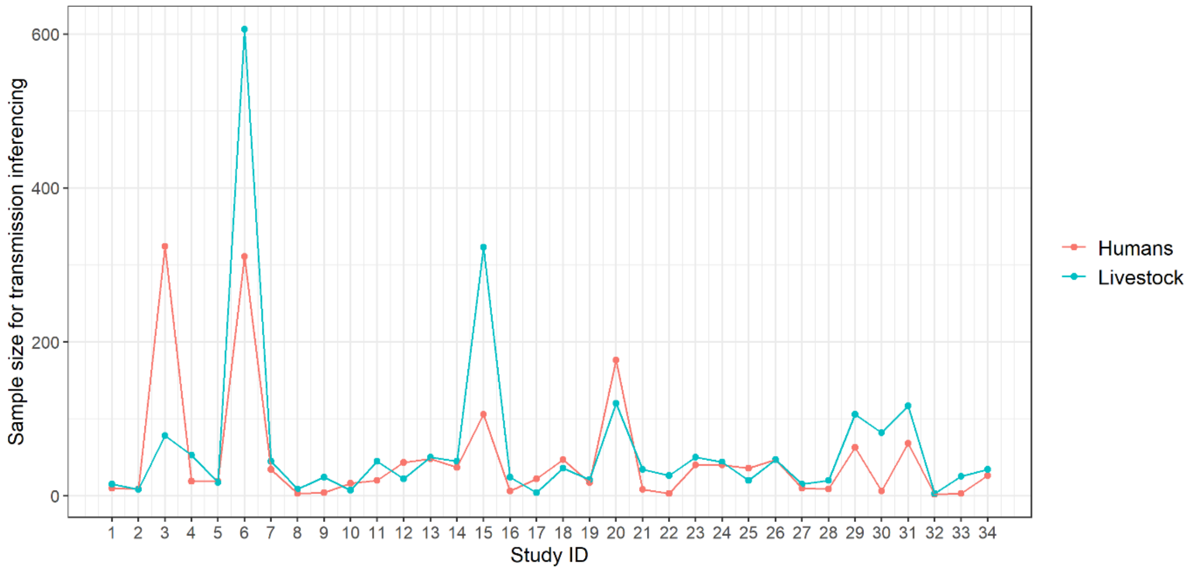

Supplementary Figure 6: Number of human and livestock samples used for AMR transmission inferencing at the human-livestock interface.

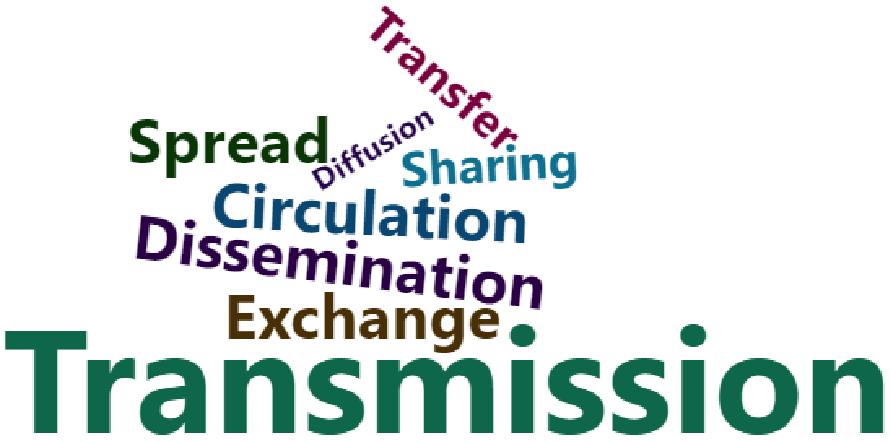

Supplementary Figure 7: Keywords used in transmission inferences. Font size represents relative frequencies.

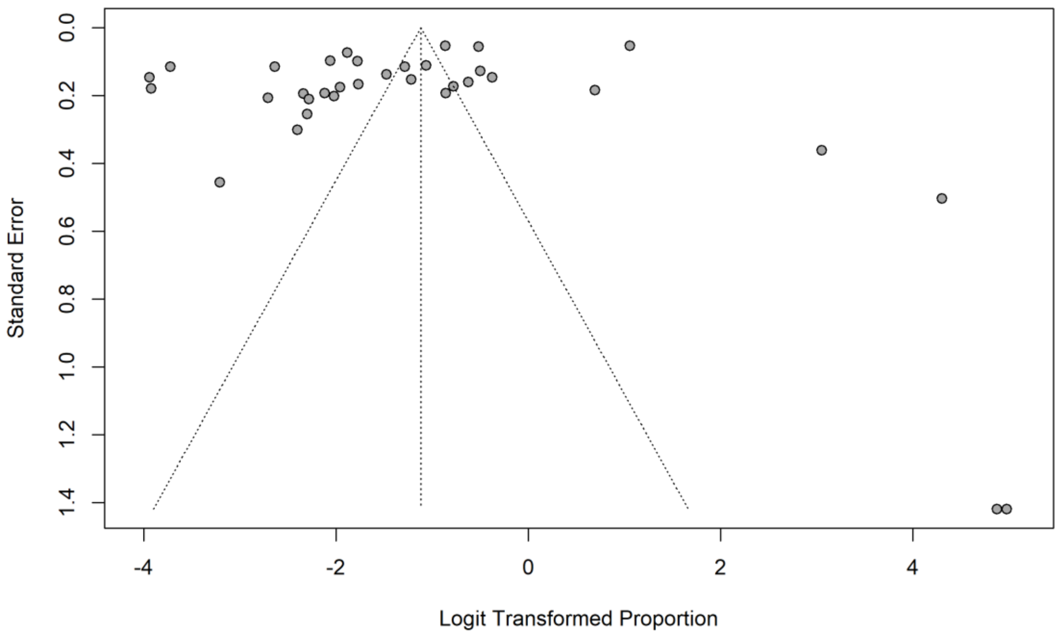

Supplementary Figure 8: Funnel plot of standard error vs. logit-transformed proportions for 34 included studies assessing the proportion of collected samples used for AMR transmission inferencing. This figure demonstrates an asymmetrical shape. While publication bias could be one of the possibilities, this could also reflect small-study effect or true heterogeneity rather than publication bias.

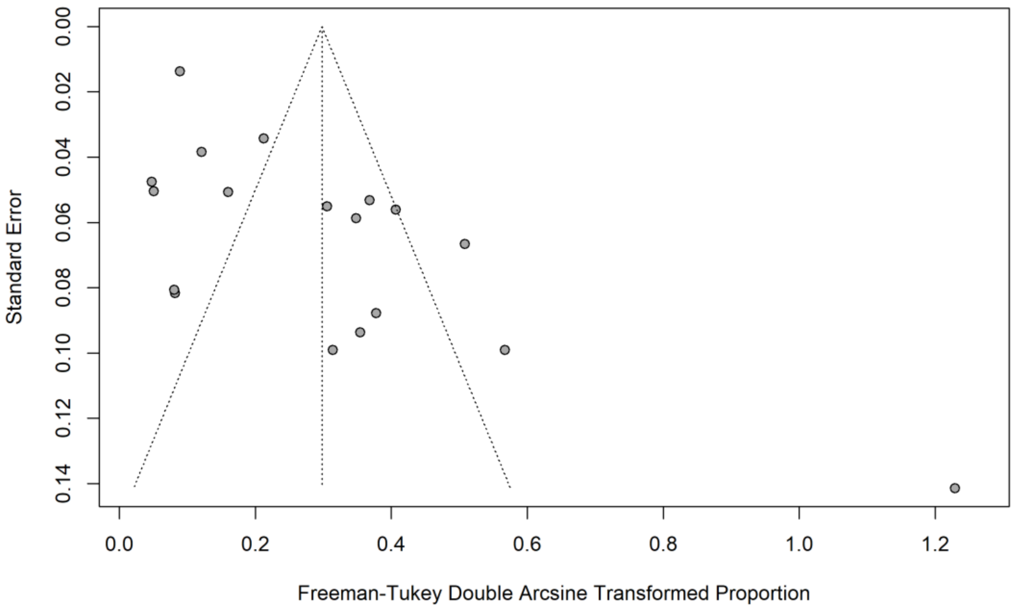

Supplementary Figure 9: Funnel plot of standard error vs. logit-transformed proportions for 18 included studies assessing the proportion of individuals involved in AMR transmission at the human-livestock interface. The funnel plot is asymmetrical. While publication bias is one of the possibilities, this could also reflect small-study effect or true heterogeneity rather than publication bias

Supplementary Table 1: Species of bacteria reported in included studies

Gram stain classification (number of publications)	Bacteria species (number of publications)	Resistance determinants encoded
Gram-negative (24)	*Escherichia coli* (19)	*IncF* plasmids [22,25] ; *IncL* [25] ; *blaCTX-M-15* [18,20,33,35] ; *blaCTX-M-14* [18] ; *blaCTX-M-1*[51,52] ; *blaOXA-48* [23] ; *blaOXA-181* [35] ; *blaTEM-1* [35,52] ; *blaSHV* [53]; *blaIMP* [52]; *ColKP3-IncX3* [35]; *gyrA* & *parC* [35]; *aph(3")−Ib*, *aph(6)−Id*, *dfrA14*, *sul2*, *qnrS1* & *blaEC* [35]
	Klebsiella pneumoniae (6)	*BlaOXA-48* (Mairi et al., 2019) ; *blaCTX-M-15* [34]
	Klebsiella oxitoca (2)	-
	Salmonella enterica (4)	*aac(3)-Id*; *aadA7*, *tetA*, *sul1*, *parC*, *gyrA* [31]; *blaTEM-1* [31,54]; plasmid *Col440I_1* [29]
	Other Gram-negative species*: Acinetobacter baumannii, Proteus mirabilis, serratiafonticola, Kluyvera ascorbate, Enterobacter cloacae, Enterobacter aerogenes, Raoultella ornithinolytica, Cronobacter malonaticus, Citrobacter werkmanii, Pluralibacter gergoviae* (3)	-

Gram positive (7)	*Staphylococcus aureus* (5)	*blaZ* gene [30,32] *mecA* [19,40] SCC*mec* [30]
	Coagulase-negative staphylococci -CoNS: *Staphylococcus hominis, Staphylococcus lugdunensis, Staphylococcus scuiri, Staphylococcus simulans, Staphylococcus epidermidis, Staphylococcus chromogens, Staphylococcus haemolyticus, Staphylococcus xylosus, Staphylococcus cohnii, Staphylococcus condiment, Staphylococcus arlettae, Staphylococcus equorum, Staphylococcus saprophyticus, Staphylococcus lentus, Staphylococcus vitulinus* (3)	*SCCmec* [55] ; *mecA* [19]
	Other Gram-positive species: *Enterococci* (1).	-

Supplementary Table 2: Summary information of the 31 studies included

ID	Author (Year of publication) [Country]	Title	Study design, interface, location, period and scope	Transmission inference and direction	Link to paper
1	Egyir et al. (2020) [Ghana]	Whole-genome sequence profiling of antibiotic-resistant *Staphylococcus aureus* isolates from livestock and farm attendants in Ghana	This cross-sectional study was conducted at the human-livestock interface in southern Ghana in 2017. The study used whole genome sequencing (WGS) to provide baseline information on antimicrobial resistance, population structure, and virulence gene content of *S. aureus* isolates from livestock and farm attendants.	The study inferred transmission of AMR bacteria between humans and goats. The inferred direction of transmission was human to goat.	https://dx.doi.org/10.1016/j.jgar.2020.03.029
2	Madoshi et al. (2016) [Tanzania]	Characterisation of Commensal *Escherichia coli* Isolated from Apparently Healthy Cattle and Their Attendants in Tanzania	This cross-sectional study was conducted at the human-cattle interface in urban and peri-urban locations of Morogoro, Tanzania in 2014. Enterobacteria Intragenic Consensus - Polymerase chain reaction (ERIC-PCR) fingerprinting and WGS were used to analyse AMR and genetic relatedness of E.coli populations from apparently healthy cattle and their attendants in cattle farms, and investigate pathogenic potential of commensal *E. coli* and possible exchange of the genomic content between cattle and human isolates.	The study concluded exchange of antimicrobial and virulence gene carrying plasmids between cattle and their attendants. The direction of transmission was not specified.	https://dx.doi.org/10.1371/journal.pone.0168160

3	Weiss et al. (2018) [Uganda]	Antibiotic-Resistant *Escherichia coli* and Class 1 Integrons in Humans, Domestic Animals, and Wild Primates in Rural Uganda	This cross-sectional study was implemented in 2005 in the rural areas of western Uganda, near Kibale national park and Bwindi impenetrable national park. Samples were collected at the human, livestock and wildlife interface. The study's objective was to study antibiotic resistance and class 1 integrons in *E.coli* using disc diffusion and PCR.	The study inferred dissemination/diffusion of antibiotic resistant bacteria and resistance-conferring genetic elements between geographical locations and among humans, livestock and wild animals. The direction of transmission was not specified.	https://dx.doi.org/10.1128/AEM.01632-18
4	Dieye et al. (2022) [Senegal]	Genomics of human and chicken *Salmonella* isolates in Senegal: Broilers as a source of antimicrobial resistance and potentially invasive nontyphoidal salmonellosis infections	This cross-sectional study was conducted at the human-poultry interface in Dakar, Senegal between July 2012 and June 2013. The aim of this study was to determine the resistance profile of nontyphoidal *Salmonella* strains from clinical salmonellosis cases in humans, and the potential transmission of these strains from retail chicken meat.	The study suggested possible transmission of the emerging multidrug resistant (MDR) Salmonella Kentucky ST198 and serotype Schwarzengrund from chicken to human	https://dx.doi.org/10.1371/journal.pone.0266025

5	Akinyemi et al. (2023) [Nigeria]	Whole genome sequencing of *Salmonella enterica* serovars isolated from humans, animals, and the environment in Lagos, Nigeria	This cross-sectional study was implemented at a human-livestock-environment interface in urban Nigeria between December 2017 and May 2019. Samples were collected from human patients, livestock from markets, and waste water. The aim of this study was to investigate virulence and antimicrobial resistance genes among the Salmonella enterica serovars and to identify potential clonal relationships between strains from different sources using whole genome sequencing.	This study inferred transmission of antimicrobial resistance genes carrying *Salmonella enterica* serovars at the human-livestock-environment interface. There were no explicit inferences about the direction of transmission.	https://dx.doi.org/10.1186/s12866-023-02901-1
6	Muloi et al. (2022) [Kenya]	Population genomics of *Escherichia coli* in livestock-keeping households across a rapidly developing urban landscape	This cross-sectional study targeting synanthropic wildlife and sympatric human and livestock populations in the urban areas of Nairobi, Kenya, was carried out from August 2015 to October 2016. The objective of the study was to identify risk factors to help inform surveillance strategies that target potential hotspots for strain sharing and AMR transmission among populations in an urban setting.	This study reported sharing of *E.coli* and AMR genes between humans and livestock within and between households. The direction of sharing was not specified.	https://dx.doi.org/10.1038/s41564-022-01079-y

7	Falgenhauer et al. (2019) [Ghana]	Detection and Characterization of ESBL-Producing *Escherichia coli* From Humans and Poultry in Ghana	This cross-sectional study was conducted between January and June 2015 at the human-poultry interface (Children admitted at a hospital and poultry farms) in Ghana. This study aimed to compare ESBL-producing *E. coli* found in the intestinal tract of humans and poultry using highly discriminatory WGS methods in order to assess potential transmission routes in a rural community of Central Ghana.	The study inferred clonal transmission of ESBL-Producing *Escherichia coli* From Humans to Poultry in Ghana.	https://dx.doi.org/10.3389/fmicb.2018.03358
8	Amoako et al. (2019) [South Africa]	Genomic analysis of methicillin-resistant *Staphylococcus aureus* isolated from poultry and occupational farmworkers in Umgungundlovu District, South Africa	This was a cross-sectional study conducted at the human-poultry interface in uMgungundlovu District in KwaZulu-Natal Province, South Africa. The study implementation period was not indicated. This study investigated the antibiotic resistome, MGEs and genetic lineages of circulating MRSA isolated from poultry and occupationally exposed workers in an intensive poultry production system using WGS.	This study concluded that there was transmission of MDR- MRSA between humans and poultry within the poultry value chain. The inferred direction of transmission was from human to poultry.	https://dx.doi.org/10.1016/j.scitotenv.2019.03.110

9	Mairi et al. (2019) [Algeria]	OXA-48-producing *Enterobacterales* in different ecological niches in Algeria: clonal expansion, plasmid characteristics and virulence traits	This cross-sectional study was conducted to investigate the prevalence and molecular characteristics of OXA-48-carbapenemase-producing *Enterobacterales* strains recovered from the human-livestock-environment interface in Algeria using repetitive sequence-based PCR (Rep-PCR) and MLST. The study was conducted between December 2015 and April 2017.	The study suggested a wide dissemination of OXA-48-producing *Enterobacterales* among different ecological niches (Humans, animals, food, environment). The direction of transmission was not inferred.	https://dx.doi.org/10.1093/jac/dkz146
10	Mohamed et al. (2023) [Djibouti]	Genomic epidemiology of carbapenemase-producing Gram-negative bacteria at the human-animal-environment interface in Djibouti city, Djibouti	This cross-sectional study used WGS to assess CP-GNB circulation at the human-livestock-environment interface in Djibouti City. The study period was between 1 January and 31 July 2019.	The study reported circulation of CP-GNB (Carbapenemase producing *E. coli* ST10) and plasmids harbouring carbapenem resistance at the human-animal-environment interface. The direction of transmission was not specified.	https://dx.doi.org/10.1016/j.scitotenv.2023.167160

11	Ahmed et al. (2023) [Egypt]	Extended-spectrum B-lactamase-producing *E. coli* from retail meat and workers: genetic diversity, virulotyping, pathotyping and the antimicrobial effect of silver nanoparticles	This cross-sectional study was conducted between January and March 2022 to determine the occurrence, pathotypes, virulotypes, genotypes, and antimicrobial resistance patterns of ESBL-producing *E. coli* in retail meat samples and workers in retail meat shops in Egypt using REP-PCR, and to evaluate the bactericidal efficacy of silver nanoparticles (AgNPs-H2O2) against multidrug resistant (MDR) ESBL-producing *E. coli.*	The study inferred transmission of ESBL bacteria from animal products to humans.	https://dx.doi.org/10.1186/s12866-023-02948-0
12	Abd El-Hamid et al. (2018) [Egypt]	What is behind phylogenetic analysis of hospital, community and livestock associated methicillin resistant *Staphylococcus aureus*	This cross-sectional study was conducted between June 2016 and March 2017 in Zagazig city, Egypt at the interface of humans and livestock. The objective of the study was to (a) identify and compare the currently available phenotypic antimicrobial resistance profiles as well as the genotypic features of HA-, CA- and LA-MRSA strains in Egypt, (b) determine if there were a phenotypic or genotypic relationship between the MRSA strains isolated from the same geographical area.	The study inferred a bidirectional transmission/ dissemination of methicillin resistant *Staphylococcus aureus.*	https://dx.doi.org/10.1111/tbed.13170

13	Ramadan et al. (2018) [Egypt]	Contribution of Healthy Chickens to Antimicrobial-Resistant *Escherichia coli* Associated with Human Extraintestinal Infections in Egypt.	This cross-sectional study was conducted between June and December 2015 in Ismailia, sharkia and Dakahlia governorates, Egypt. The study aimed to determine the coexistence of antimicrobial resistance, virulence-associated genes, and phylogenetic groups *in E. coli* isolates from patients with extraintestinal infections and healthy chickens. Pulsed-field gel electrophoresis (PFGE) was used to determine the clonality of isolates.	This study concluded that there was no transmission of AMR between the human and poultry population.	https://dx.doi.org/10.1089/vbz.2017.2237
14	Djeffal et al. (2017) [Algeria]	Prevalence and clonal relationship of ESBL producing *Salmonella* strains from humans and poultry in northeastern Algeria	This cross-sectional study was conducted between December 2011 and May 2013 in north eastern Algeria at the interface of humans and poultry. The aims of this study were to investigate *Salmonella* contamination in broiler chicken farms and slaughterhouses, to assess the antibiotic resistance profile in avian and human Salmonella isolates, and to evaluate the relationship between avian and human Extended Spectrum β-Lactamase (ESBL)-producing isolates.	This study inferred transmission of ESBL-producing *Salmonella* from poultry to humans.	https://dx.doi.org/10.1186/s12917-017-1050-3

15	Gay et al. (2023) [Madagascar]	One Health compartment analysis of ESBL-producing *Escherichia coli* reveals multiple transmission events in a rural area of Madagascar	This cross-sectional study was implemented April to October 2018 (dry season), in Andoharanofotsy, Madagascar at the human-livestock-companion animal-environment interface. It used WGS to characterize ESBL-*E. coli* genomic diversity within and between human, animal and environmental compartments in a suburban rural area of Madagascar.	The study inferred transmission of ESBL producing *E.coli* between humans and livestock. Inferences about the direction of transmission were not made.	https://dx.doi.org/10.1093/jac/dkad125
16	Adefioye et al. (2021) [Nigeria]	Phylogenetic Characterization and Multilocus Sequence Typing of Extended-Spectrum Beta Lactamase-Producing Escherichia coli from Food-Producing Animals, Beef, and Humans in Southwest Nigeria	This cross-sectional study was conducted to investigated the resistance patterns and phylogenetic relatedness of E. coli isolates from humans, food-producing animals, and beef in Osun and Oyo States, Southwestern Nigeria, between July 2015 and June 2016.	Transmission of ESBL-producing E.coli was inferred in this study.	https://dx.doi.org/10.1089/mdr.2019.0397

17	Katakweba et al. (2015) [Tanzania]	Spa typing and antimicrobial resistance of *Staphylococcus aureus* from healthy humans, pigs and dogs in Tanzania	This cross-sectional study was conducted at the human-livestock-companion animal interface in urban and peri-urban areas of Morogoro Municipality, Tanzania, from December 2011 through March 2012. The aim of the study was to investigate the prevalence of S. aureus nasal carriage in healthy humans, pigs and dogs in Tanzania, by characterizing *S. aureus* using spa gene typing and antimicrobial resistance among human and veterinary isolates.	This study concluded that was no transmission of AMR between humans and pigs.	https://dx.doi.org/10.3855/jidc.6790
18	Aworh et al. (2021) [Nigeria]	Genetic relatedness of multidrug resistant *Escherichia coli* isolated from humans, chickens and poultry environments	The cross-sectional study was conducted to investigate the genetic relatedness of MDR *E. coli* isolates from poultry-workers, chickens, and selected poultry farms/live bird markets environments in Abuja, Nigeria. Data was collected from December 2018 to February 2020.	This study inferred that there was no clonal spread of MDR *E. coli* at the human-animal-environment interface.	https://dx.doi.org/10.1186/s13756-021-00930-x
19	Kateete et al. (2013) [Uganda]	Prevalence and Antimicrobial Susceptibility Patterns of Bacteria from Milkmen and Cows with Clinical Mastitis in and	This cross-sectional study was conducted at the human-cattle interface to describe the distribution and antimicrobial susceptibility patterns of bacteria from cows with clinical mastitis in Kampala, Uganda. It was	The study concluded that there was no transmission of AMR between humans and animals.	https://dx.doi.org/10.1371/journal.pone.0063413

		around Kampala, Uganda	implemented between February 2010 through March 2011.		
20	Iramiot et al. (2020) [Uganda]	Antimicrobial resistance at the human–animal interface in the Pastoralist Communities of Kasese District, South Western Uganda	This cross-sectional study was conducted at the human-cattle interface in the pastoralist communities of Kasese District, South Western Uganda between January 2018 and March 2019. The objective was to assess antimicrobial use and to describe factors associated with transmission of antimicrobial resistance between humans and animals in pastoralist communities.	The study concluded that there was transmission of AMR bacteria between humans and cattle. Inferences about direction of transmission were not made.	https://doi.org/10.1038/s41598-020-70517-w
21	Founou et al. (2018) [Cameroon]	Genome Sequencing of Extended-Spectrum b-Lactamase (ESBL)-Producing *Klebsiella pneumoniae* Isolated from Pigs and Abattoir Workers in Cameroon	The cross-sectional study was conducted from March to October 2016 in Cameroon at the human-pig interface. The aim was to investigate the antibiotic resistance genes, virulence factors, MGEs and genetic lineages of circulating ESBL-producing *K. pneumoniae* strains isolated from pigs and exposed workers in Cameroonian abattoirs using whole genome sequencing (WGS), to ascertain zoonotic transmission (viz. from animals to humans and/or vice-	The study inferred zoonotc transmission of ESBL-producing *K. pneumoniae*. The direction of transmission was not inferred.	https://dx.doi.org/10.3389/fmicb.2018.00188

			versa) of ESBL-producing K. pneumoniae in the food chain.		
22	Egyir et al. (2022) [Ghana]	Antimicrobial resistance and genomic analysis of *staphylococci* isolated from livestock and farm attendants in Northern Ghana	This cross-sectional study was conducted at the human-livestock interface in Northern Ghana in July 2018. The objective of the study was to characterize *Staphylococci* recovered from livestock and farm attendants using phenotypic and genotypic methods.	Transmission of AMR bacteria was inferred at the human-livestock interface. The direction of transmission was not inferred.	https://dx.doi.org/10.1186/s12866-022-02589-9
23	Lupindu et al. (2015) [Tanzania]	Transmission of antibiotic-resistant *Escherichia coli* between cattle, humans and the environment in peri-urban livestock keeping communities in Morogoro, Tanzania	This cross-sectional study was implemented in the peri-urban livestock keeping communities in Morogoro, Tanzania at the human-cattle-environment interface between December 2010 and February 2012. The aim of the study was to determine whether the close human-livestock proximity farming systems now widely practiced in many developing countries confer risk of pathogen transmission and which factors are associated with such risk.	The study reported transfer of AMR *E.coli* between cattle and humans and the environment. The direction of transmission was not inferred.	https://dx.doi.org/10.1016/j.prevetmed.2014.12.005

24	Aworh et al. (2022) [Nigeria]	Extended-Spectrum ß-Lactamase-Producing *Escherichia coli* Among Humans, Beef Cattle, and Abattoir Environments in Nigeria	This cross-sectional study investigated the zoonotic transmission of extended-spectrum beta-lactamase-producing *E. coli* (ESBL-EC) among humans, beef cattle, and abattoir environments in Abuja and Lagos, Nigeria. It was carried out from May 2020 to December 2020.	The study inferred transmission of ESBL-Producing *Escherichia coli* between humans and cattle. No inferences were made about the direction of transmission.	https://dx.doi.org/10.3389/fcimb.2022.869314
25	Shawa et al. (2022) [Zambia]	Clonal relationship between multidrug-resistant *Escherichia coli* ST69 from poultry and humans in Lusaka, Zambia	This cross-sectional study was conducted in Lusaka, Zambia in 2018 at the human-poultry interface. Its objective was to investigate the potential dissemination of MDR *E. coli* between poultry and humans in Zambia.	The study inferred that there is transmission of MDR *E.coli* between humans and poultry. No inferences were made about the direction of transmission.	https://dx.doi.org/10.1093/femsle/fnac004
26	Afema et al. (2016) [Uganda]	Potential Sources and Transmission of *Salmonella* and Antimicrobial Resistance in Kampala, Uganda	This cross-sectional study was implemented at the human-livestock-environment interface in Kampala, Uganda between 2012 and 2013. The objective was to investigate potential sources of non–typhoidal *Salmonella* in Kampala, Uganda by determining occurrence in human, livestock and environmental sources, and analyse AMR and genotypic	This study suggested that resistance determinants or resistant bacteria were disseminated across different one health compartments.	https://dx.doi.org/10.1371/journal.pone.0152130

			structure in common serovars in order to infer transmission.		
27	Elmowalid et al. (2018) [Egypt]	Molecular Detection of New SHV B-lactamase Variants in Clinical *Escherichia coli* and *Klebsiella pneumoniae* Isolates from Egypt	This cross-sectional study was implemented at the interface of humans and poultry in different localities across Egypt between October 2015 and September 2017. The objective of this study was to investigate AMR, and determine genetic relationships of MDR *E. coli* and *K. pneumoniae* isolates recovered from human subjects, day-old chicks and broiler clinical samples.	The study inferred that there was transfer of AMR genes (blaSHV genes) between human and poultry *E.coli* isolates. The direction of transmission was not inferred in the study.	https://dx.doi.org/10.1016/j.cimid.2018.09.013
28	Dhaouadi et al. (2020) [Tunisia]	Prevalence of meticillin-resistant and -susceptible coagulase-negative *staphylococci* with the first detection of the mecC gene among cows, humans and manure in Tunisia	The objective of this cross-sectional study were to assess coagulase-negative *staphylococci* (CNS) from cows, humans and manure as reservoirs of the mecA and mecC genes, and to evaluate the genetic relatedness of CNS from different origins by pulsed-field gel electrophoresis (PFGE). It was conducted between April 2015 and May 2016 in north-east Tunisia.	This study inferred interspecies dissemination of antibiotic resistance determinants between humans and cattle. The direction of transmission was not inferred.	https://dx.doi.org/10.1016/j.ijantimicag.2019.10.007

29	Strasheim et al. (2024) [South Africa]	Whole-Genome Sequencing of Human and Porcine *Escherichia coli* Isolates on a Commercial Pig Farm in South Africa	This cross-sectional study was conducted in North West province of South Africa in December 2019 at the human-pig interface. The purpose of the study was to describe and compare *E. coli* isolates obtained from pigs and human contacts from a commercial farm using conventional methods and whole-genome sequencing (WGS).	The study inferred transmission and antibiotic resistance genes between humans and pigs. The direction of transmission was not inferred.	https://dx.doi.org/10.3390/antibiotics13060543
30	Matakone et al. (2024) [Cameroon]	Multi-drug resistant (MDR) and extended-spectrum β-lactamase (ESBL) producing *Escherichia coli* isolated from slaughtered pigs and slaughterhouse workers in Yaoundé, Cameroon.	This cross-sectional study was conducted to determine the prevalence, antimicrobial resistance profiles, resistance genes and clonal relatedness of MDR and ESBL *E.coli* isolated from slaughtered pigs and exposed workers in two selected pig slaughterhouses in Yaound´e, Cameroon. The implementation period was February to May 2023.	Cross-transmission of Multi-drug resistant and extended-spectrum β-lactamase (ESBL) producing Escherichia coli between pigs and slaughter house workers was inferred. The direction of transmission was not specified.	https://dx.doi.org/10.1016/j.onehlt.2024.100885

31	Milenkov et al. (2024) [Madagascar]	Implementation of the WHO Tricycle protocol for surveillance of extended-spectrum β-lactamase producing *Escherichia coli* in humans, chickens, and the environment in Madagascar: a prospective genomic epidemiology study.	The objective of this study was to assess ESBL-*E. coli* prevalence and describe intrasector and intersector circulation of ESBL-E coli and plasmids at the human-poultry interface. This study was conducted in Antananarivo, Madagascar and samples were cross-sectionally collected between May 2018 to April 2019.	Circulation of ESBL E.coli and ESBL-carrying plasmids between humans and poultry was inferred. The direction of transmission was not inferred.	https://dx.doi.org/10.1016/S2666-5247(24)00065-X
32	Badr et al. (2022) [Egypt]	Multidrug-Resistant and Genetic Characterization of Extended-Spectrum Beta-Lactamase-Producing *E. coli* Recovered from Chickens and Humans in Egypt	The aim of this study was to investigate the phenotypic and genotypic characteristics of ESBL-producing E. coli samples obtained from diseased chickens in farms from northern part of Egypt and samples retrieved from human cases from Qena, Egypt. This cross-sectional study was conducted between 2019 and 2020.	The study inferred transmission of ESBL E. coli from chicken to humans without specifying the basis on which the direction of transmission was inferred.	https://dx.doi.org/10.3390/ani12030346
33	Eldesoukey et al. (2022) [Egypt]	Multidrug-Resistant Enteropathogenic *Escherichia coli* Isolated from Diarrhoeic Calves, Milk, and Workers in	The objectives of this cross-sectional study were to (1) to investigate the distribution frequency of EPEC isolates from diarrhoeic calves, milk, and workers in dairy farms, (2) to	The study inferred transmission of MDR-EPEC and/or their AMR genes at the human dairy cattle interface.	https://dx.doi.org/10.3390/antibiotics11080999

		Dairy Farms: A Potential Public Health Risk	record the antibiogram of isolates and their associated AMR genetic determinants, and (3) to assess the genetic relatedness between isolates using repetitive extragenic palindromic sequence-based PCR (REP-PCR) for evidence of potential infection pathways within farms. This study was conducted in Egypt in 2019.	The direction of transmission was not inferred.	
34	Sadat et al. (2022)	Phylotypic Profiling, Distribution of Pathogenicity Island Markers, and Antimicrobial Susceptibility of *Escherichia coli* Isolated from Retail Chicken Meat and Humans	This cross-sectional study was conducted in Egypt between January and July 2019 in Egypt at the human-chicken interface. The aim of the study was to evaluate *E. coli* strains for phylogenetic profiles, antimicrobial resistance, presence of PAI markers, and biofilm formation from chickens and humans, as well as to analyse the link among the existence of the PAIs and the phylogenetic groups from both sources.	The study inferred transmission of antibiotic resistance genes from poultry to humans or humans to humans through the food chain. The basis on which the bidirectional transmission was based was not specified.	https://dx.doi.org/10.3390/antibiotics11091197

Supplementary Table 3: PRISMA 2020 Main Checklist

Topic	No.	Item	Location where item is reported
**TITLE**			
**Title**	1	Identify the report as a systematic review.	Title
**ABSTRACT**			
**Abstract**	2	See the PRISMA 2020 for Abstracts checklist	Page 2
**INTRODUCTION**			
**Rationale**	3	Describe the rationale for the review in the context of existing knowledge.	Pages 3-4
**Objectives**	4	Provide an explicit statement of the objective(s) or question(s) the review addresses.	Page 4
**METHODS**			
**Eligibility criteria**	5	Specify the inclusion and exclusion criteria for the review and how studies were grouped for the syntheses.	Pages 20-21
**Information sources**	6	Specify all databases, registers, websites, organisations, reference lists and other sources searched or consulted to identify studies. Specify the date when each source was last searched or consulted.	Page 20
**Search strategy**	7	Present the full search strategies for all databases, registers and websites, including any filters and limits used.	Page 20
**Selection process**	8	Specify the methods used to decide whether a study met the inclusion criteria of the review, including how many reviewers screened each record and each report retrieved, whether they worked independently, and if applicable, details of automation tools used in the process.	Page 20-21

Topic	No.	Item	Location where item is reported
**Data collection process**	9	Specify the methods used to collect data from reports, including how many reviewers collected data from each report, whether they worked independently, any processes for obtaining or confirming data from study investigators, and if applicable, details of automation tools used in the process.	Page 21
**Data items**	10a	List and define all outcomes for which data were sought. Specify whether all results that were compatible with each outcome domain in each study were sought (e.g. for all measures, time points, analyses), and if not, the methods used to decide which results to collect.	Page 21
	10b	List and define all other variables for which data were sought (e.g. participant and intervention characteristics, funding sources). Describe any assumptions made about any missing or unclear information.	Page 21
**Study risk of bias assessment**	11	Specify the methods used to assess risk of bias in the included studies, including details of the tool(s) used, how many reviewers assessed each study and whether they worked independently, and if applicable, details of automation tools used in the process.	Page 21-22
**Effect measures**	12	Specify for each outcome the effect measure(s) (e.g. risk ratio, mean difference) used in the synthesis or presentation of results.	Pages 10 and 12
**Synthesis methods**	13a	Describe the processes used to decide which studies were eligible for each synthesis (e.g. tabulating the study intervention characteristics and comparing against the planned groups for each synthesis (item 5)).	Page 12

Topic	No.	Item	Location where item is reported
	13b	Describe any methods required to prepare the data for presentation or synthesis, such as handling of missing summary statistics, or data conversions.	Pages 21-22
	13c	Describe any methods used to tabulate or visually display results of individual studies and syntheses.	Pages 21-22
	13d	Describe any methods used to synthesize results and provide a rationale for the choice(s). If meta-analysis was performed, describe the model(s), method(s) to identify the presence and extent of statistical heterogeneity, and software package(s) used.	Pages 21-22
	13e	Describe any methods used to explore possible causes of heterogeneity among study results (e.g. subgroup analysis, meta-regression).	Pages 22
	13f	Describe any sensitivity analyses conducted to assess robustness of the synthesized results.	n/a
**Reporting bias assessment**	14	Describe any methods used to assess risk of bias due to missing results in a synthesis (arising from reporting biases).	n/a
**Certainty assessment**	15	Describe any methods used to assess certainty (or confidence) in the body of evidence for an outcome.	Supplementary Figure 1
**RESULTS**			
**Study selection**	16a	Describe the results of the search and selection process, from the number of records identified in the search to the number of studies included in the review, ideally using a flow diagram.	Fig. 2

Topic	No.	Item	Location where item is reported
	16b	Cite studies that might appear to meet the inclusion criteria, but which were excluded, and explain why they were excluded.	Fig. 2
**Study characteristics**	17	Cite each included study and present its characteristics.	Supplementary Table 2
**Risk of bias in studies**	18	Present assessments of risk of bias for each included study.	Supplementary Fig 1; page 5
**Results of individual studies**	19	For all outcomes, present, for each study: (a) summary statistics for each group (where appropriate) and (b) an effect estimate and its precision (e.g. confidence/credible interval), ideally using structured tables or plots.	Supplementary Table 2
**Results of syntheses**	20a	For each synthesis, briefly summarise the characteristics and risk of bias among contributing studies.	Page 5
	20b	Present results of all statistical syntheses conducted. If meta-analysis was done, present for each the summary estimate and its precision (e.g. confidence/credible interval) and measures of statistical heterogeneity. If comparing groups, describe the direction of the effect.	Pages 9-10; 12; Supplementary Figures 8 & 9
	20c	Present results of all investigations of possible causes of heterogeneity among study results.	Page 9; Supplementary Figure 8 & 9
	20d	Present results of all sensitivity analyses conducted to assess the robustness of the synthesized results.	n/a
**Reporting biases**	21	Present assessments of risk of bias due to missing results (arising from reporting biases) for each synthesis assessed.	n/a
**Certainty of evidence**	22	Present assessments of certainty (or confidence) in the body of evidence for each outcome assessed.	Supplementary Figure 1

Topic	No.	Item	Location where item is reported
**DISCUSSION**			
**Discussion**	23a	Provide a general interpretation of the results in the context of other evidence.	Pages 13-16
	23b	Discuss any limitations of the evidence included in the review.	Pages 13-16
	23c	Discuss any limitations of the review processes used.	13 -16
	23d	Discuss implications of the results for practice, policy, and future research.	Pages 16-19
**OTHER INFORMATION**			
**Registration and protocol**	24a	Provide registration information for the review, including register name and registration number, or state that the review was not registered.	Page 19
	24b	Indicate where the review protocol can be accessed, or state that a protocol was not prepared.	Page 19
	24c	Describe and explain any amendments to information provided at registration or in the protocol.	n/a
**Support**	25	Describe sources of financial or non-financial support for the review, and the role of the funders or sponsors in the review.	n/a
**Competing interests**	26	Declare any competing interests of review authors.	Page 28

From: Page MJ, McKenzie JE, Bossuyt PM, Boutron I, Hoffmann TC, Mulrow CD, et al. The PRISMA 2020 statement: an updated guideline for reporting systematic reviews. MetaArXiv. 2020, September 14. DOI: 10.31222/osf.io/v7gm2. For more information, visit: www.prisma-statement.org
